# Multi-criteria decision making in robotic agri-farming with q-rung orthopair m-polar fuzzy sets

**DOI:** 10.1371/journal.pone.0246485

**Published:** 2021-02-25

**Authors:** Muhammad Riaz, Muhammad Tahir Hamid, Deeba Afzal, Dragan Pamucar, Yu-Ming Chu

**Affiliations:** 1 Department of Mathematics, University of the Punjab, Lahore, Lahore, Pakistan; 2 Department of Mathematics & Statistics, The University of Lahore, Lahore, Pakistan; 3 Department of Logistics, University of Defence, Belgrade, Serbia; 4 Department of Mathematics, Huzhou University, Huzhou, P. R. China; Universita degli Studi del Molise, ITALY

## Abstract

q-Rung orthopair fuzzy set (qROFS) and m-polar fuzzy set (mPFS) are rudimentary concepts in the computational intelligence, which have diverse applications in fuzzy modeling and decision making under uncertainty. The aim of this paper is to introduce the hybrid concept of q-rung orthopair m-polar fuzzy set (qROmPFS) as a hybrid model of q-rung orthopair fuzzy set and m-polar fuzzy set. A qROmPFS has the ability to deal with real life situations when decision experts are interested to deal with multi-polarity as well as membership and non-membership grades to the alternatives in an extended domain with q-ROF environment. Certain operations on qROmPFSs and several new notions like support, core, height, concentration, dilation, *α*-cut and (*α*, *β*)-cut of qROmPFS are defined. Additionally, grey relational analysis (GRA) and choice value method (CVM) are presented under qROmPFSs for multi-criteria decision making (MCDM) in robotic agri-farming. The proposed methods are suitable to find out an appropriate mode of farming among several kinds of agri-farming. The applications of proposed MCDM approaches are illustrated by respective numerical examples. To justify the feasibility, superiority and reliability of proposed techniques, the comparison analysis of the final ranking in the robotic agri-farming computed by the proposed techniques with some existing MCDM methods is also given.

## 1 Introduction

A dynamic and strategic approach to decision making plays a very important role in providing the right decision at the right time and right place. Multi-criteria decision making (MCDM) is a tool that provides harmony in the ranking of alternative under multiple criterion. MCDM provides a feasible decision by the decision makers while considering a set multiple criterion which help them in the ranking of short listed alternatives that fulfill their requirements and seek an optimal alternative. The awareness with these methods is very much essential for efficacious and systematized decision making. MCDM has been intensively studied by numerous researchers. The techniques developed for this task mainly depend on the type of decision problem under consideration. The problem of imperfect, uncertain and vague information has been the focus of many researchers for the last decades. In order to deal with such problems, Zadeh [1] initiated the idea of a fuzzy set with the help of membership function. Subsequently, the intuitionistic fuzzy set (IFS) was proposed by Atanassov [2] as an extension of the fuzzy set by means of the membership and the non-membership functions. Soft set theory introduced by Molodtsov [3] to deal with vague information and modeling uncertainty. Yager [4, 5] introduced Pythagorean fuzzy set (PFS) as an extension of Atanassov’s intuitionistic fuzzy set. Yager [6] further introduced the concept of q-rung orthopair fuzzy sets (qROFSs). A qROFS is the generalization of both IFS and PFS. The main feature of qROFS is that the uncertain space for membership grades and non-memberships grades is boarder.

Fuzzy sets and it extensions like IFSs, PFSs and q-ROFSs have been studied by many researchers; Ali *et al*. [7], Garg [8, 11], Feng *et al*. [12], Hashmi *et al*. [13], Hashmi and Riaz [14], Karaaslan [15], Karaaslan and Hunu [16], Naeem *et al*. [17], Peng and Yang [18], Peng *et al*. [19], Peng and Selvachandran [20], Peng and Liu [21], Riaz and Hashmi [22] and Riaz *et al*. [23, 24].

A strong MCDM approach named as TOPSIS “technique for ordering preference through the ideal solution” have been fascinated by numerous researchers; Akram and Adeel [25], Chen [26], Chen and Tsao [27], Dey *et al*. [28], Eraslan and Karaaslan [29], Kumar and Garg [30], Li and Nan [31], Selvachandran and Peng [32], Tehrim and Riaz [33] and Zhang and Xu [34]. Zhang [35] introduced bipolar fuzzy sets as extension of fuzzy sets in 1994. Lee [36], in 2000, presented an extension of fuzzy sets named as bipolar-valued fuzzy sets and presented two kinds of its representation. Chen *et al*. [37] generalized the notion of bipolar fuzzy sets to m-polar fuzzy sets and rendered some applications of m-polar fuzzy sets in real world problems. Some extension of fuzzy sets have studied by many researchers [38–46].

Huang *et al*. [47] introduced Pythagorean fuzzy MULTIMOORA method based on distance measure and score function: its application in multi-criteria decision-making process. Hussain *et al*. [48] explored rough Pythagorean fuzzy ideals in semigroups. Jan *et al*. [49] proposed an approach towards decision making and shortest path problems using the concepts of interval-valued Pythagorean fuzzy information. Lin *et al*. [50] introduced various decision making methods including Linguistic q-rung orthopair fuzzy sets and their interactional partitioned Heronian mean aggregation operators. Lin *et al*. [51] introduced TOPSIS method based on correlation coefficient and entropy measure for linguistic Pythagorean fuzzy sets and its application to multiple attribute decision making (MADM). Lin *et al*. [52] proposed an evaluating IoT platforms using integrated probabilistic linguistic MCDM Method. Lin *et al*. [53] explored MULTIMOORA based MCDM model for site selection of car sharing station under picture fuzzy environment.

Riaz and Tehrim [54, 55] introduced geometric aggregation operators under cubic bipolar fuzzy sets. They presented a robust extension of VIKOR method for bipolar fuzzy sets using connection numbers of SPA theory based metric spaces. Ullah *et al*. [56] introduced some distance measures of complex Pythagorean fuzzy sets and their applications in pattern recognition. Wei [57, 58] introduced gray relational analysis (GRA) method for intuitionistic fuzzy multiple attribute decision making.

The first goal of the paper is to introduce q-rung orthopair m-polar fuzzy set as a hybrid model of q-rung orthopair fuzzy set and m-polar fuzzy set. A qROmPFS is a new approach towards uncertainty which is superior to existing approaches of intuitionistic m-polar fuzzy sets and Pythagorean m-polar fuzzy sets. The eminent characteristic of qROmPFS is that it deals with the real life situation when multi-polarity of membership and non-membership grades is necessary to each alternative in a larger uncertain space with q-ROF environment. When *m* = 1, this model becomes a q-rung orthopair fuzzy set and q-rung orthopair bipolar fuzzy set for *m* = 2. As qROFS is superior approach towards uncertainty than intuitionistic fuzzy set (IFS) and Pythagorean fuzzy set (PFS), So the proposed model of qROmPFS is superior approach than both intuitionistic m-polar fuzzy set (IMPFS) and Pythagorean m-polar fuzzy set (PMPFS).

The second goal of this paper is to develop a robust MCDM approach with q-rung orthopair m-polar fuzzy information in which various uncertainties can be considered by q-rung orthopair m-polar fuzzy numbers. In order to find an optimal decision, an optimization model of grey relational analysis (GRA) and generalized choice value method (GCVM) under qROmPFSs are developed and illustrated by the respective numerical examples.

To facilitate our discussion, the remaining article is arranged as follows: In Section 2, the rudimentary concepts of fuzzy sets, soft sets, IFSs, PFSs and qROFSs are given that would be helpful in the study of this research work. Section 3 of this article introduces the novel concepts of q-rung orthopair m-polar fuzzy sets along with some associated operations on qROmPFSs and their related results. The concepts of *α*-cut and (*α*, *β*)-cut of qROmPFSs are also defined. In Section 4, the extension of GRA and GCVM to qROmPFSs are established for MCDM in the robotic agri-farming. The application of the proposed MCDM approaches is illustrated by the respective numerical examples and well justified by comparison analysis with some existing techniques. We summarized this research work with a concrete conclusion in Section 5.

## 2 Preliminaries

This section provides a review of some rudimentary concepts of PFSs, qROFSs and mPFSs that will be helpful for better understanding of the current research work. In 2014, Yager [4, 5] proposed the notion of PFS as an extension of IFS. So that every intuitionistic fuzzy number (IFN) is a Pythagorean fuzzy number (PFN) but not conversely. So a PFN is superior to IFN.

**Definition 2.1** [4, 5] A Pythagorean fuzzy set, P shortly written (PFS), upon *X* is represented in a well known format,

$$P=\{<\xi ,\mu_{P}\left(\xi \right),\nu_{P}\left(\xi \right)>:\xi \in X\}$$

where $\mu_{P}\left(\xi \right)\in \left[0,1\right]$ and $\nu_{P}\left(\xi \right)\in \left[0,1\right]$ are the membership and non-membership degrees, respectively, such that the sum of their squares should not go beyond unity. The ordered pair so obtained $\left(\mu_{P},\nu_{P}\right)$ is called Pythagorean fuzzy number (PFN). The value $\gamma_{P}\left(\xi \right)=\sqrt{1-\mu_{P}^{2}\left(\xi \right)-\nu_{P}^{2}\left(\xi \right)}$ is called degree of hesitancy.

In 2017, Yager [6] introduced the idea of q-rung orthopair fuzzy set (qROFS), which is the next generation of IFS and PFS.

**Definition 2.2** [6] Let *X* be the universal set. A q-rung orthopair fuzzy set (qROFS) with *q* > 1 can be defined as

$$R=\{<\xi ,\mu_{R}\left(\xi \right),\nu_{R}\left(\xi \right)>:\;0\leq {\left(\mu_{R}\left(\xi \right)\right)}^{q}+{\left(\nu_{R}\left(\xi \right)\right)}^{q}\leq 1,\xi \in X\}$$

where *μ*_*R*_(*ξ*) ∈ [0, 1] and *ν*_*R*_(*ξ*) ∈ [0, 1] represent the degree of membership and the degree of non-membership of *ξ* ∈ *X*.

In general,

$${ϒ}_{R}=\sqrt[{q}]{1-{\left(\mu_{R}\left(\xi \right)\right)}^{q}-{\left(\nu_{R}\left(\xi \right)\right)}^{q}}$$

is known as the degree of hesitancy for *ξ* to *R*. The pair (*μ*_*R*_(*ξ*), *ν*_*R*_(*ξ*)), for each *ξ* ∈ *X*, is said to be a q-rung orthopair fuzzy number (qROFN). By definition of qROFS, we simply say that qROFS can be split into a classes of orthopair fuzzy numbers with distinct values of *q*. For example, when *q* = 1 it becomes intuitionistic fuzzy number (IFN) and when *q* = 2 it becomes Pythagorean fuzzy number (PFN). Thus IFN and PFN are special cases of qROFN.

**Definition 2.3** [37] An m-polar fuzzy set (or [0, 1]^*m*^-set) belonging to a reference set *X*, designated by a mapp *A*: *X* ↦ [0, 1]^*m*^, for a natural number *m*. The set of all m-polar fuzzy sets on *X* is denoted by *m*(*X*).

## 3 q-Rung orthopair m-polar fuzzy sets

In this section, we discuss the innovative hybrid structure named as q-rung orthopair m-polar fuzzy set (qROmPFS). When *m* = 1, this model becomes a q-rung orthopair fuzzy set and q-rung orthopair bipolar fuzzy set for *m* = 2. For *q* = 2 it reduces to Pythagorean m-polar fuzzy sets and becomes a PFS for *q* = 2 and *m* = 1.

**Definition 3.1** *A q-rung orthopair m-polar fuzzy set* R, *abbreviated as qROmPFS, on universal set X is characterized with the help of a mapp* $\mu_{R}^{\left(j\right)}:X\to \left[0,1\right]$ *(called membership functions) and* $\nu_{R}^{\left(j\right)}:X\to \left[0,1\right]$ *(called non-membership functions) fulfilling the condition as the entirety (sum) of their squared values ought to not surpass unity i.e*. $0\leq {\left(\mu_{R}^{\left(j\right)}\left(\xi \right)\right)}^{q}+{\left(\nu_{R}^{\left(j\right)}\left(\xi \right)\right)}^{q}\leq 1$, (*q* > 1) for *j* = 1, 2, ⋯, *m*, *for a natural number m*.

*A qROmPFS can be written as* $R=\{\langle \xi ,((\mu_{R}^{\left(1\right)}\left(\xi \right),\nu_{R}^{\left(1\right)}\left(\xi \right)),\cdots ,(\mu_{R}^{\left(m\right)}\left(\xi \right),\nu_{R}^{\left(m\right)}\left(\xi \right)))\rangle :0\leq {\left(\mu_{R}^{\left(j\right)}\left(\xi \right)\right)}^{q}+{\left(\nu_{R}^{\left(j\right)}\left(\xi \right)\right)}^{q}\leq 1,\xi \in X\}$ *or in alternative ways*

$$\begin{array}{ccc} R & = & \left\{\frac{\xi }{\left((\mu_{R}^{\left(1\right)}\left(\xi \right),\nu_{R}^{\left(1\right)}\left(\xi \right)),\cdots ,(\mu_{R}^{\left(m\right)}\left(\xi \right),\nu_{R}^{\left(m\right)}\left(\xi \right))\right)}:\xi \in X\right\}\\ & = & \left\{\frac{\xi }{\left((\mu_{R}^{\left(j\right)}\left(\xi \right),\nu_{R}^{\left(j\right)}\left(\xi \right))\right)}:\xi \in X;j=1,2,\cdots ,m\right\} \end{array}$$

*Let us further assume that X* = {*ξ*_1_, *ξ*_2_, ⋯ *ξ*_*r*_} *i.e. the cardinality of X is r*, *the tabular form of qROmPFS* R *is given in* Table 1 *and the matrix format is as follows*.

$$\begin{array}{c} R=[\begin{array}{cccc} \left(\mu_{R}^{\left(1\right)}\left(\xi_{1}\right),\nu_{R}^{\left(1\right)}\left(\xi_{1}\right)\right) & \left(\mu_{R}^{\left(2\right)}\left(\xi_{1}\right),\nu_{R}^{\left(2\right)}\left(\xi_{1}\right)\right) & \cdots & \left(\mu_{R}^{\left(m\right)}\left(\xi_{1}\right),\nu_{R}^{\left(m\right)}\left(\xi_{1}\right)\right)\\ \left(\mu_{R}^{\left(1\right)}\left(\xi_{2}\right),\nu_{R}^{\left(1\right)}\left(\xi_{2}\right)\right) & \left(\mu_{R}^{\left(2\right)}\left(\xi_{2}\right),\nu_{R}^{\left(2\right)}\left(\xi_{2}\right)\right) & \cdots & \left(\mu_{R}^{\left(m\right)}\left(\xi_{2}\right),\nu_{R}^{\left(m\right)}\left(\xi_{2}\right)\right)\\ \vdots & \vdots & \ddots & \vdots \\ \left(\mu_{R}^{\left(1\right)}\left(\xi_{r}\right),\nu_{R}^{\left(1\right)}\left(\xi_{r}\right)\right) & \left(\mu_{R}^{\left(2\right)}\left(\xi_{r}\right),\nu_{R}^{\left(2\right)}\left(\xi_{r}\right)\right) & \cdots & \left(\mu_{R}^{\left(m\right)}\left(\xi_{r}\right),\nu_{R}^{\left(m\right)}\left(\xi_{r}\right)\right) \end{array}] \end{array}$$

*This r* × *m matrix is mentioned as qROmPF-matrix*.

**Table 1:** Tabular form of qROmPFS R.

R				
*ξ*_1_	$\left(\mu_{R}^{\left(1\right)}\left(\xi_{1}\right),\nu_{R}^{\left(1\right)}\left(\xi_{1}\right)\right)$	$\left(\mu_{R}^{\left(2\right)}\left(\xi_{1}\right),\nu_{R}^{\left(2\right)}\left(\xi_{1}\right)\right)$	⋯	$\left(\mu_{R}^{\left(m\right)}\left(\xi_{1}\right),\nu_{R}^{\left(m\right)}\left(\xi_{1}\right)\right)$
*ξ*_2_	$\left(\mu_{R}^{\left(1\right)}\left(\xi_{2}\right),\nu_{R}^{\left(1\right)}\left(\xi_{2}\right)\right)$	$\left(\mu_{R}^{\left(2\right)}\left(\xi_{2}\right),\nu_{R}^{\left(2\right)}\left(\xi_{2}\right)\right)$	⋯	$\left(\mu_{R}^{\left(m\right)}\left(\xi_{2}\right),\nu_{R}^{\left(m\right)}\left(\xi_{2}\right)\right)$
⋮	⋮	⋮	⋱	⋮
*ξ*_*r*_	$\left(\mu_{R}^{\left(1\right)}\left(\xi_{r}\right),\nu_{R}^{\left(1\right)}\left(\xi_{r}\right)\right)$	$\left(\mu_{R}^{\left(2\right)}\left(\xi_{r}\right),\nu_{R}^{\left(2\right)}\left(\xi_{r}\right)\right)$	⋯	$\left(\mu_{R}^{\left(m\right)}\left(\xi_{r}\right),\nu_{R}^{\left(m\right)}\left(\xi_{r}\right)\right)$

*If we collect all qROmPFSs defined over X then this may be written as qROmPFS*(*X*).

**Example 3.2** *We consider a classical set X* = {*f*, *g*}, *then*

$$R=\{\frac{f}{\left(0.315,0.271),(0.710,0.143),(0.287,0.614\right)},\frac{g}{\left(0.517,0.352),(0.273,0.619),(0.403,0.516\right)}\}$$

*is a q-rung orthopair 3-polar fuzzy set (qRO3PFS). Its matrix form may be expressed as*

$$\begin{array}{c} R=[\begin{array}{ccc} \left(0.315,0.271\right) & \left(0.710,0.143\right) & \left(0.287,0.614\right)\\ \left(0.517,0.352\right) & \left(0.273,0.619\right) & \left(0.403,0.516\right) \end{array}] \end{array}$$

### 3.1 Operations on q-rung orthopair fuzzy sets

**Definition 3.3** *Let* R *be the qROmPFS on X*. *Then the collection of the points ξ of X for which* $\mu_{R}^{\left(j\right)}\left(\xi \right)>0$ or $\nu_{R}^{\left(j\right)}\left(\xi \right)<1$, *for minimum one value of* 1 ≤ *j* ≤ *m*, *is said to be support of* R *i.e*.

$$supp\left(R\right)=\{\xi \in X:\mu_{R}^{\left(j\right)}\left(\xi \right)>0\;or\;\nu_{R}^{\left(j\right)}\left(\xi \right)<1\;for\;at\;least\;one\;j=1,2,\cdots ,m\}$$

**Definition 3.4** *Suppose that* R *be a qROmPFS over X*. *The collection of the points ξ* ∈ *X where* $\mu_{R}^{\left(j\right)}\left(\xi \right)=1$ (*so manifestly* $\nu_{R}^{\left(j\right)}\left(\xi \right)=0$), *for at least one j* = 1, 2, ⋯, *m*, *is said to be core of* R *i.e*.

$$core\left(R\right)=\{\xi \in X:\mu_{R}^{\left(j\right)}\left(\xi \right)=1\;for\;at\;least\;one\;j=1,2,\cdots ,m\}$$

*We exemplify the notions of support and core in the example given below*.

**Example 3.5** *For the qRO3PFS described over X* = {*d*, *f*, *g*, *h*}, *for which*

$$\begin{array}{c} R=[\begin{array}{ccc} \left(0.345,0.012\right) & \left(0.493,0.131\right) & \left(0.511,0.179\right)\\ \left(0.419,0.007\right) & \left(0.700,0.243\right) & \left(0.432,0.145\right)\\ \left(1.000,0.000\right) & \left(0.666,0.200\right) & \left(0.811,0.009\right)\\ \left(0.000,0.100\right) & \left(0.000,0.100\right) & \left(0.000,0.100\right) \end{array}] \end{array}$$

we have

supp(R)={d,f,g}

core(R)={g}

**Definition 3.6** *We consider a set* R *a qROmPFS over* X. *The highest value which the membership function* $\mu_{R}^{\left(j\right)}\left(\xi \right)$, *obtained for all* ξ∈X *where* 1 ≤ *j* ≤ *m*}, *is named as height of* R *and is termed as* ht(R). *A qROmPFS* R *is called normal if* ht(R)=1 *and is said to be subnormal on the other hand*.

**Example 3.7** *For* R *presented in Example 3.2*,

ht(R)=0.710

*for* R *presented in Example 3.5*,

ht(R)=1

*Here*, R *shown in Example 3.5 is normal while qROmPFS* R *shown in Example 3.2 is subnormal*.

**Definition 3.8** *We consider two qROmPFSs* $R_{1}$ *and* $R_{2}$ *over X*. *We consider* $R_{1}$ *as a subset of* $R_{2}$, *written* $R_{1}⊑R_{2}$, *if* $\mu_{R_{1}}^{\left(j\right)}\left(\xi \right)\leq \mu_{R_{2}}^{\left(j\right)}\left(\xi \right)$ *and* $\nu_{R_{1}}^{\left(j\right)}\left(\xi \right)\geq \nu_{R_{2}}^{\left(j\right)}\left(\xi \right)$, *for all ξ* ∈ *X and for all permissible values of j*.

$R_{1}$ *and* $R_{2}$ *are known as equal providing that one of these values is intervened in between the others i.e*. $R_{1}⊑R_{2}⊑R_{1}$.

**Example 3.9** *Let*

$$\begin{array}{c} R_{1}=[\begin{array}{ccc} \left(0.453,0.224\right) & \left(0.365,0.372\right) & \left(0.725,0.411\right)\\ \left(0.753,0.413\right) & \left(0.444,0.266\right) & \left(0.837,0.284\right)\\ \left(0.810,0.110\right) & \left(0.004,0.072\right) & \left(0.100,1.040\right) \end{array}] \end{array}$$

*and*

$$\begin{array}{c} R_{2}=[\begin{array}{ccc} \left(0.555,0.111\right) & \left(0.672,0.228\right) & \left(0.768,0.161\right)\\ \left(0.774,0.228\right) & \left(0.599,0.134\right) & \left(0.872,0.231\right)\\ \left(0.871,0.102\right) & \left(0.038,0.019\right) & \left(0.218,0.026\right) \end{array}] \end{array}$$

*be qROmPFSs over X*, *and we can say that* $R_{1}⊑R_{2}$.

**Remark 1** *We consider the two qROmPFSs* $R_{1}$ *and* $R_{2}$ *over* *X*, *and* $R_{1}⊑R_{2}$, *this implies that* $ht\left(R_{1}\right)\leq ht\left(R_{2}\right)$. *Its converse may not be true*.

**Definition 3.10** *A qROmPFS* R *upon* *X* *is called null qROmPFS if* $\mu_{R}^{\left(j\right)}\left(\xi \right)=0$ *and* $\nu_{R}^{\left(j\right)}\left(\xi \right)=1$, *for each* *ξ* ∈ *X* *and all permissible values of j*, *and is represented by* Φ. *Thus*,

$$\Phi =[\begin{array}{cccc} \left(0,1\right) & \left(0,1\right) & \cdots & \left(0,1\right)\\ \left(0,1\right) & \left(0,1\right) & \cdots & \left(0,1\right)\\ \vdots & \vdots & \ddots & \vdots \\ \left(0,1\right) & \left(0,1\right) & \cdots & \left(0,1\right) \end{array}]$$

*Here support and core of* Φ *are null set, as well as the height of* Φ *is zero, thus* Φ *is subnormal qROmPFS*.

**Definition 3.11** *A qROmPFS* R *over X is known as absolute qROmPFS if* $\mu_{R}^{\left(j\right)}\left(\xi \right)=1$ *and* $\nu_{R}^{\left(j\right)}\left(\xi \right)=0$, *for every ξ* ∈ *X and all permissible values of j*. *It is represented by* Ψ. *Thus*,

$$\Psi =[\begin{array}{cccc} \left(1,0\right) & \left(1,0\right) & \cdots & \left(1,0\right)\\ \left(1,0\right) & \left(1,0\right) & \cdots & \left(1,0\right)\\ \vdots & \vdots & \ddots & \vdots \\ \left(1,0\right) & \left(1,0\right) & \cdots & \left(1,0\right) \end{array}]$$

*The support and core of* Ψ *is X*, *the height of* Ψ *is 1*, *thus* Ψ *is normal qROmPFS*.

**Proposition 3.12** *If* R *is any qROmPFS over X*, *then* Φ⊑R⊑Ψ.

**Proof 1** *The proof follows by definitions of null set* Φ *and absolute* Ψ.

**Definition 3.13** *Let* R *be a qROmPFS given by*

$$\begin{array}{ccc} R & = & \left\{\frac{\xi }{\left((\mu_{R}^{\left(j\right)}\left(\xi \right),\nu_{R}^{\left(j\right)}\left(\xi \right))\right)}:\xi \in X;j=1,2,\cdots ,m\right\} \end{array}$$

*then its complement is defined by*

$$\begin{array}{ccc} R^{c} & = & \left\{\frac{\xi }{\left((\nu_{R}^{\left(j\right)}\left(\xi \right),\mu_{R}^{\left(j\right)}\left(\xi \right))\right)}:\xi \in X;j=1,2,\cdots ,m\right\} \end{array}$$

*Notice that* Φ^*c*^ = Ψ and Ψ^*c*^ = Φ. *Moreover*, ${\left(R^{c}\right)}^{c}=R$.

**Example 3.14** *Let* R *be the qROmPFS in matrix form*

$$\begin{array}{c} R=[\begin{array}{ccc} \left(0.315,0.271\right) & \left(0.710,0.143\right) & \left(0.287,0.614\right)\\ \left(0.517,0.352\right) & \left(0.273,0.619\right) & \left(0.403,0.516\right) \end{array}] \end{array}$$

*then its complement may be represented as*

$$R^{c}=[\begin{array}{ccc} \left(0.271,0.315\right) & \left(0.143,0.710\right) & \left(0.614,0.287\right)\\ \left(0.352,0.517\right) & \left(0.619,0.273\right) & \left(0.516,0.403\right) \end{array}]$$

**Definition 3.15** *The union of two qROmPFSs* $R_{1}$ *and* $R_{2}$ *discussed upon the same universal set X is represented as*

$$R_{1}⊔R_{2}=\{\frac{\xi }{\left(\max(\mu_{R_{1}}^{\left(i\right)}\left(\xi \right),\mu_{R_{2}}^{\left(j\right)}\left(\xi \right)),\min(\nu_{R_{1}}^{\left(j\right)}\left(\xi \right),\nu_{R_{2}}^{\left(j\right)}\left(\xi \right))\right)}:\xi \in X;1\leq j\leq m\}$$

**Definition 3.16** *Let* $R_{1}$ *and* $R_{2}$ *be qROmPFSs upon the same universal set* X. *Their intersection may be discussed as*:

$$R_{1}⊓R_{2}=\{\frac{\xi }{\left(\min(\mu_{R_{1}}^{\left(i\right)}\left(\xi \right),\mu_{R_{2}}^{\left(j\right)}\left(\xi \right)),\max(\nu_{R_{1}}^{\left(j\right)}\left(\xi \right),\nu_{R_{2}}^{\left(j\right)}\left(\xi \right))\right)}:\xi \in X;j=1,2,\cdots ,m\}$$

**Example 3.17** *We consider a classical set Let* X={f,g,h}, *also*

$$\begin{array}{ccc} R_{1} & = & \left[\begin{array}{cccc} \left(0.531,0.222\right) & \left(0.412,0.204\right) & \left(0.555,0.301\right) & \left(0.156,0.870\right)\\ \left(0.831,0.231\right) & \left(0.732,0.444\right) & \left(0.830,0.010\right) & \left(0.812,0.110\right)\\ \left(0.766,0.244\right) & \left(0.456,0.140\right) & \left(0.571,0.473\right) & \left(0.611,0.142\right) \end{array}\right]\\ R_{2} & = & \left[\begin{array}{cccc} \left(0.514,0.345\right) & \left(0.819,0.009\right) & \left(0.700,0.227\right) & \left(0.153,0.625\right)\\ \left(0.712,0.106\right) & \left(0.513,0.300\right) & \left(0.729,0.115\right) & \left(0.822,0.200\right)\\ \left(0.632,0.301\right) & \left(1.000,0.000\right) & \left(0.768,0.072\right) & \left(0.000,1.000\right) \end{array}\right] \end{array}$$

*then*

$$\begin{array}{ccc} R_{1}⊔R_{2} & = & \left[\begin{array}{cccc} \left(0.531,0.222\right) & \left(0.819,0.009\right) & \left(0.700,0.227\right) & \left(0.156,0.625\right)\\ \left(0.831,0.106\right) & \left(0.732,0.300\right) & \left(0.830,0.010\right) & \left(0.822,0.110\right)\\ \left(0.766,0.244\right) & \left(1.000,0.000\right) & \left(0.768,0.072\right) & \left(0.611,0.142\right) \end{array}\right]\\ R_{1}⊓R_{2} & = & \left[\begin{array}{cccc} \left(0.514,0.345\right) & \left(0.412,0.204\right) & \left(0.555,0.301\right) & \left(0.153,0.870\right)\\ \left(0.712,0.231\right) & \left(0.513,0.444\right) & \left(0.729,0.115\right) & \left(0.812,0.200\right)\\ \left(0.632,0.301\right) & \left(0.456,0.140\right) & \left(0.571,0.473\right) & \left(0.000,1.000\right) \end{array}\right] \end{array}$$

**Proposition 3.18** *If* R, $R_{1}$, $R_{2}$ *and* $R_{3}$ *are qROmPFSs over X*, *then*

- Φ⊔R=R.
- Φ⊓R=Φ.
- Ψ⊔R=Ψ.
- Ψ⊓R=R.
- R⊔R=R.
- R⊓R=R.
- $R_{1}⊔R_{2}=R_{2}⊔R_{1}$.
- $R_{1}⊓R_{2}=R_{2}⊓R_{1}$.
- $R_{1}⊔\left(R_{2}⊔R_{3}\right)=\left(R_{1}⊔R_{2}\right)⊔R_{3}$.
- $R_{1}⊓\left(R_{2}⊓R_{3}\right)=\left(R_{1}⊓R_{2}\right)⊓R_{3}$.
- $R_{1}⊔\left(R_{2}⊓R_{3}\right)=\left(R_{1}⊔R_{2}\right)⊓\left(R_{1}⊔R_{3}\right)$.
- $R_{1}⊓\left(R_{2}⊔R_{3}\right)=\left(R_{1}⊓R_{2}\right)⊔\left(R_{1}⊓R_{3}\right)$.

**Proof 2** *The proof is obvious*.

**Corollary 3.19**

- Φ ⊔ Ψ = Ψ.
- Φ ⊓ Ψ = Φ.

**Proposition 3.20** *If* $R_{1}$ *and* $R_{2}$ *are qROmPFSs over X*, *then any one of them may be sandwiched between* $R_{1}⊓R_{2}$ *and* $R_{1}⊔R_{2}$ *i.e*.

- $R_{1}⊓R_{2}⊑R_{1}$ *and* $R_{1}⊑R_{1}⊔R_{2}$.
- $R_{1}⊓R_{2}⊑R_{2}$ *and* $R_{2}⊑R_{1}⊔R_{2}$.

**Proof 3** *(i) It is clear that* $\min\{\mu_{R_{1}}^{\left(j\right)},\mu_{R_{2}}^{\left(j\right)}\}\leq \mu_{R_{1}}^{\left(j\right)}\leq \max\{\mu_{R_{1}}^{\left(j\right)},\mu_{R_{2}}^{\left(j\right)}\}$ *and* $\max\{\nu_{R_{1}}^{\left(j\right)},\nu_{R_{2}}^{\left(j\right)}\}\geq \nu_{R_{1}}^{\left(j\right)}\geq \min\{\nu_{R_{1}}^{\left(j\right)},\nu_{R_{2}}^{\left(j\right)}\}$. *Similarly we can obtain(ii)*.

**Proposition 3.21** *De Morgan laws hold for* $R_{1}$ *and* $R_{2}$ *in qROmPFSs over X*, *i.e*.

- ${\left(R_{1}⊔R_{2}\right)}^{c}=R_{1}^{c}⊓R_{2}^{c}$.
- ${\left(R_{1}⊓R_{2}\right)}^{c}=R_{1}^{c}⊔R_{2}^{c}$.

**Proof 4** *For the proof of these laws, we consider only the 1st law. Second De Morgan law may be proved with the same working*. *Here we assume that* $\max(\mu_{R_{1}}^{\left(j\right)}\left(\xi \right),\mu_{R_{2}}^{\left(j\right)}\left(\xi \right))=\mu_{R_{1}}^{\left(j\right)}\left(\xi \right)$ *also* $\max(\nu_{R_{1}}^{\left(j\right)}\left(\xi \right),\nu_{R_{2}}^{\left(j\right)}\left(\xi \right))=\nu_{R_{1}}^{\left(j\right)}\left(\xi \right)$. *So for each* *ξ* ∈ *X* and *j* = 1, 2, ⋯, *m*

$$\begin{array}{ccc} {\left(R_{1}⊔R_{2}\right)}^{c} & = & {\left\{\frac{\xi }{\left(\max(\mu_{R_{1}}^{\left(j\right)}\left(\xi \right),\mu_{R_{2}}^{\left(j\right)}\left(\xi \right)),\min(\nu_{R_{1}}^{\left(j\right)}\left(\xi \right),\nu_{R_{2}}^{\left(j\right)}\left(\xi \right))\right)}\right\}}^{c}\\ & = & {\left\{\frac{\xi }{\left(\mu_{R_{1}}^{\left(j\right)}\left(\xi \right),\nu_{R_{2}}^{\left(j\right)}\left(\xi \right)\right)}\right\}}^{c}\\ & = & \left\{\frac{\xi }{\left(\nu_{R_{2}}^{\left(j\right)}\left(\xi \right),\mu_{R_{1}}^{\left(j\right)}\left(\xi \right)\right)}\right\}\\ & = & \left\{\frac{\xi }{\left(\min(\nu_{R_{1}}^{\left(j\right)}\left(\xi \right),\nu_{R_{2}}^{\left(j\right)}\left(\xi \right)),\max(\mu_{R_{1}}^{\left(j\right)}\left(\xi \right),\mu_{R_{2}}^{\left(j\right)}\left(\xi \right))\right)}\right\}\\ & = & \left\{\frac{\xi }{\left((\nu_{R_{1}}^{\left(j\right)}\left(\xi \right),\mu_{R_{1}}^{\left(j\right)}\left(\xi \right))\right)}\right\}⊓\{\frac{\xi }{\left((\nu_{R_{2}}^{\left(j\right)}\left(\xi \right),\mu_{R_{2}}^{\left(j\right)}\left(\xi \right))\right)}\}\\ & = & {\left\{\frac{\xi }{\left((\mu_{R_{1}}^{\left(j\right)}\left(\xi \right),\nu_{R_{1}}^{\left(j\right)}\left(\xi \right))\right)}\right\}}^{c}⊓{\left\{\frac{\xi }{\left((\mu_{R_{2}}^{\left(j\right)}\left(\xi \right),\nu_{R_{2}}^{\left(j\right)}\left(\xi \right))\right)}\right\}}^{c}\\ & = & R_{1}^{c}⊓R_{2}^{c} \end{array}$$

**Example 3.22** *Let* R *be the qROmPFS in matrix form*

$$\begin{array}{c} R=[\begin{array}{ccc} \left(0.315,0.271\right) & \left(0.710,0.143\right) & \left(0.287,0.614\right)\\ \left(0.517,0.352\right) & \left(0.273,0.619\right) & \left(0.403,0.516\right) \end{array}] \end{array}$$

*then its compliment may be represented as*

$$R^{c}=\left[\begin{array}{ccc} \left(0.271,0.315\right) & \left(0.143,0.710\right) & \left(0.614,0.287\right)\\ \left(0.352,0.517\right) & \left(0.619,0.273\right) & \left(0.516,0.403\right) \end{array}\right]$$

$$\begin{array}{ccc} R⊔R^{c} & = & \left[\begin{array}{c} \left\{(0.315,0.271),(0.710,0.143),(0.614,0.287)\right\}\\ \left\{(0.517,0.352),(0.619,0.273),(0.516,0.403)\right\} \end{array}\right]\\ & \neq & \Psi \end{array}$$

*and*

$$\begin{array}{ccc} R⊓R^{c} & = & \left[\begin{array}{c} \left\{(0.271,0.315),(0.143,0.710),(0.287,0.614)\right\}\\ \left\{(0.352,0.517),(0.273,0.619),(0.403,0.516)\right\} \end{array}\right]\\ & \neq & \Phi \end{array}$$

**Proposition 3.23** *Let* R *be the qROmPFS on* X, *then*

- $R⊔R^{c}\neq \Psi$.
- $R⊓R^{c}\neq \Phi$.

**Definition 3.24** *Let* $R_{1}$ *and* $R_{2}$ *be qROmPFSs from a universal set* X, *their difference may be represented as*:

$$R_{1}\backslash R_{2}=\left\{\frac{\xi }{\left(\max(\mu_{R_{1}}^{\left(j\right)}\left(\xi \right),\nu_{R_{2}}^{\left(j\right)}\left(\xi \right)),\min(\nu_{R_{1}}^{\left(j\right)}\left(\xi \right),\mu_{R_{2}}^{\left(j\right)}\left(\xi \right))\right)}:\xi \in X;j=1,2,\cdots ,m\right\}$$

**Example 3.25** *Let*

$$\begin{array}{ccc} R_{1} & = & \left[\begin{array}{cccc} \left(0.531,0.222\right) & \left(0.412,0.204\right) & \left(0.555,0.301\right) & \left(0.156,0.870\right)\\ \left(0.831,0.231\right) & \left(0.732,0.444\right) & \left(0.830,0.010\right) & \left(0.812,0.110\right)\\ \left(0.766,0.244\right) & \left(0.456,0.140\right) & \left(0.571,0.473\right) & \left(0.611,0.142\right) \end{array}\right]\\ R_{2} & = & \left[\begin{array}{cccc} \left(0.514,0.345\right) & \left(0.819,0.009\right) & \left(0.700,0.227\right) & \left(0.153,0.625\right)\\ \left(0.712,0.106\right) & \left(0.513,0.300\right) & \left(0.729,0.115\right) & \left(0.822,0.200\right)\\ \left(0.632,0.301\right) & \left(1.000,0.000\right) & \left(0.768,0.072\right) & \left(0.000,1.000\right) \end{array}\right] \end{array}$$

*Then the difference between* *R*_1_ *and* *R*_2_ *we have*

$$\begin{array}{ccc} R_{1}\backslash R_{2} & = & \left[\begin{array}{cccc} \left(0.531,0.222\right) & \left(0.412,0.204\right) & \left(0.555,0.301\right) & \left(0.625,0.153\right)\\ \left(0.831,0.231\right) & \left(0.732,0.444\right) & \left(0.830,0.010\right) & \left(0.512,0.110\right)\\ \left(0.766,0.244\right) & \left(0.456,0.140\right) & \left(0.571,0.473\right) & \left(1.000,0.000\right) \end{array}\right] \end{array}$$

**Definition 3.26** *Let* Ψ *be a qROmPFS and* R *is extricated from* Ψ, *then we can define the necessity operator* □ *on* R *as*

$$□R=\{\frac{\xi }{\left(\mu_{R}^{\left(j\right)}\left(\xi \right),\sqrt[{q}]{1-{\left(\mu_{R}^{\left(j\right)}\left(\xi \right)\right)}^{2}}\right)}:\xi \in \Psi ;j=1,2,\cdots ,m\}$$

**Definition 3.27** *Let* Ψ *be a qROmPFS and* R *is extricated from* Ψ, *then we can define the possibility operator* ♢ *on* R *as*

$$♢R=\{\frac{\xi }{\left(\sqrt[{q}]{1-{\left(\nu_{R}^{\left(j\right)}\left(\xi \right)\right)}^{2}},\nu_{R}^{\left(j\right)}\left(\xi \right)\right)}:\xi \in \Psi ;j=1,2,\cdots ,m\}$$

**Remark 2** *The possibility operator and necessity operator discussed here can transform any qROmPFSs* R *to m-polar fuzzy set*.

**Example 3.28** *Let* R *be the qROmPFS in matrix form*

$$\begin{array}{c} R=\left[\begin{array}{ccc} \left(0.315,0.271\right) & \left(0.710,0.143\right) & \left(0.287,0.614\right)\\ \left(0.517,0.352\right) & \left(0.273,0.619\right) & \left(0.403,0.516\right) \end{array}\right] \end{array}$$

*For q* = 2 *we have*

$$\begin{array}{ccc} □R & = & \left[\begin{array}{ccc} \left(0.315,0.949\right) & \left(0.710,0.704\right) & \left(0.287,0.958\right)\\ \left(0.517,0.856\right) & \left(0.273,0.962\right) & \left(0.403,0.915\right) \end{array}\right] \end{array}$$

*and*

$$\begin{array}{ccc} ♢R & = & \left[\begin{array}{ccc} \left(0.962,0.271\right) & \left(0.989,0.143\right) & \left(0.789,0.614\right)\\ \left(0.936,0.352\right) & \left(0.785,0.619\right) & \left(0.857,0.516\right) \end{array}\right] \end{array}$$

**Proposition 3.29** *For any qROmPFS* R, □R⊑♢R.

**Proof 5** *For every* *ξ* ∈ Ψ *and for all permissible values of j*, *we have*

$$\begin{array}{ccc} 0\leq {\left(\mu_{R}^{\left(j\right)}\left(\xi \right)\right)}^{q}+{\left(\nu_{R}^{\left(j\right)}\left(\xi \right)\right)}^{q} & \leq & 1\\ \mu_{R}^{\left(j\right)}\left(\xi \right) & \leq & \sqrt[{q}]{1-{\left(\nu_{R}^{\left(j\right)}\left(\xi \right)\right)}^{q}}\\ \&\;\nu_{R}^{\left(j\right)}\left(\xi \right) & \leq & \sqrt[{q}]{1-{\left(\mu_{R}^{\left(j\right)}\left(\xi \right)\right)}^{q}} \end{array}$$

*This give us the result*.

**Corollary 3.30** *For any qROmPFS* R, *we have*

- □R⊔♢R=♢R
- □R⊓♢R=□R

**Definition 3.31** *The sum of two qROmPFSs* $R_{1}$ *and* $R_{2}$ *extricated from the crisp set X is defined as*

$$R_{1}⊕R_{2}=\left\{\frac{\xi }{\left(\sqrt[{q}]{{\left(\mu_{R_{1}}^{\left(j\right)}\left(\xi \right)\right)}^{q}+{\left(\mu_{R_{2}}^{\left(j\right)}\left(\xi \right)\right)}^{q}-{\left(\mu_{R_{1}}^{\left(j\right)}\left(\xi \right)\mu_{R_{2}}^{\left(j\right)}\left(\xi \right)\right)}^{q}},\nu_{R_{1}}^{\left(j\right)}\left(\xi \right)\nu_{R_{2}}^{\left(j\right)}\left(\xi \right)\right)}:\xi \in X;j=1,2,\cdots ,m\right\}$$

**Example 3.32** *Let X* = {*f*, *g*, *h*} *be a crisp set and*

$$\begin{array}{ccc} R_{1} & = & \left[\begin{array}{cccc} \left(0.531,0.222\right) & \left(0.412,0.204\right) & \left(0.555,0.301\right) & \left(0.156,0.870\right)\\ \left(0.831,0.231\right) & \left(0.732,0.444\right) & \left(0.830,0.010\right) & \left(0.812,0.110\right)\\ \left(0.766,0.244\right) & \left(0.456,0.140\right) & \left(0.571,0.473\right) & \left(0.611,0.142\right) \end{array}\right]\\ R_{2} & = & \left[\begin{array}{cccc} \left(0.514,0.345\right) & \left(0.819,0.009\right) & \left(0.700,0.227\right) & \left(0.153,0.625\right)\\ \left(0.712,0.106\right) & \left(0.513,0.300\right) & \left(0.729,0.115\right) & \left(0.822,0.200\right)\\ \left(0.632,0.301\right) & \left(1.000,0.000\right) & \left(0.768,0.072\right) & \left(0.000,1.000\right) \end{array}\right] \end{array}$$

*then for q* = 2

$$\begin{array}{ccc} R_{1}⊕R_{2} & = & \left[\begin{array}{cccc} \left(0.687,0.076\right) & \left(0.852,0.002\right) & \left(0.804,0.068\right) & \left(0.217,0.544\right)\\ \left(0.921,0.025\right) & \left(0.816,0.133\right) & \left(0.924,0.001\right) & \left(0.943,0.022\right)\\ \left(0.867,0.073\right) & \left(1.000,0.000\right) & \left(0.850,0.034\right) & \left(0.611,0.142\right) \end{array}\right] \end{array}$$

*And for q* = 3

$$\begin{array}{ccc} R_{1}⊕R_{2} & = & \left[\begin{array}{cccc} \left(0.643,0.077\right) & \left(0.834,0.002\right) & \left(0.769,0.068\right) & \left(0.195,0.544\right)\\ \left(0.900,0.025\right) & \left(0.780,0.133\right) & \left(0.904,0.001\right) & \left(0.926,0.022\right)\\ \left(0.838,0.073\right) & \left(1.000,0.000\right) & \left(0.822,0.034\right) & \left(0.611,0.142\right) \end{array}\right] \end{array}$$

**Definition 3.33** *The product of two qROmPFSs* $R_{1}$ *and* $R_{2}$ *extricated from the crisp set ψ is defined as*

$$R_{1}⊗R_{2}=\left\{\frac{\xi }{\left(\mu_{R_{1}}^{\left(j\right)}\left(\xi \right)\mu_{R_{2}}^{\left(j\right)}\left(\xi \right),\sqrt[{q}]{{\left(\nu_{R_{1}}^{\left(j\right)}\left(\xi \right)\right)}^{q}+{\left(\nu_{R_{2}}^{\left(j\right)}\left(\xi \right)\right)}^{q}-{\left(\nu_{R_{1}}^{\left(j\right)}\left(\xi \right)\nu_{R_{2}}^{\left(j\right)}\left(\xi \right)\right)}^{q}}\right)}:\xi \in \psi ;j=1,2,\cdots ,m\right\}$$

**Example 3.34** *We consider a classical set* X={f,g,h}, *also*

$$\begin{array}{ccc} R_{1} & = & \left[\begin{array}{cccc} \left(0.531,0.222\right) & \left(0.412,0.204\right) & \left(0.555,0.301\right) & \left(0.156,0.870\right)\\ \left(0.831,0.231\right) & \left(0.732,0.444\right) & \left(0.830,0.010\right) & \left(0.812,0.110\right)\\ \left(0.766,0.244\right) & \left(0.456,0.140\right) & \left(0.571,0.473\right) & \left(0.611,0.142\right) \end{array}\right]\\ R_{2} & = & \left[\begin{array}{cccc} \left(0.514,0.345\right) & \left(0.819,0.009\right) & \left(0.700,0.227\right) & \left(0.153,0.625\right)\\ \left(0.712,0.106\right) & \left(0.513,0.300\right) & \left(0.729,0.115\right) & \left(0.822,0.200\right)\\ \left(0.632,0.301\right) & \left(1.000,0.000\right) & \left(0.768,0.072\right) & \left(0.000,1.000\right) \end{array}\right] \end{array}$$

*then for q* = 2

$$\begin{array}{ccc} R_{1}⊗R_{2} & = & \left[\begin{array}{cccc} \left(0.273,0.403\right) & \left(0.337,0.204\right) & \left(0.389,0.371\right) & \left(0.024,0.923\right)\\ \left(0.592,0.253\right) & \left(0.376,0.519\right) & \left(0.605,0.115\right) & \left(0.668,0.227\right)\\ \left(0.484,0.381\right) & \left(0.456,0.140\right) & \left(0.439,0.477\right) & \left(0.000,1.000\right) \end{array}\right] \end{array}$$

*And for q* = 3

$$\begin{array}{ccc} R_{1}⊗R_{2} & = & \left[\begin{array}{cccc} \left(0.273,0.372\right) & \left(0.337,0.204\right) & \left(0.389,0.338\right) & \left(0.024,0.905\right)\\ \left(0.592,0.238\right) & \left(0.376,0.482\right) & \left(0.605,0.115\right) & \left(0.667,0.210\right)\\ \left(0.484,0.346\right) & \left(0.456,0.140\right) & \left(0.439,0.474\right) & \left(0.000,1.000\right) \end{array}\right] \end{array}$$

**Definition 3.35** *When* $R_{1}=R_{2}$, *then we express* $R_{1}⊗R_{1}$ *by* $R_{1}^{2}$. *so*,

$$\begin{array}{ccc} R^{2} & = & \left\{\frac{\xi }{\left({\left(\mu_{R}^{\left(j\right)}\left(\xi \right)\right)}^{2},\sqrt[{q}]{2{\left(\nu_{R}^{\left(j\right)}\left(\xi \right)\right)}^{2}-{\left(\nu_{R}^{\left(j\right)}\left(\xi \right)\right)}^{4}}\right)}:\xi \in \Psi ;j=1,2,\cdots ,m\right\}\\ & = & \left\{\frac{\xi }{\left({\left(\mu_{R}^{\left(j\right)}\left(\xi \right)\right)}^{2},\sqrt[{q}]{1-{\left(1-{\left(\nu_{R}^{\left(j\right)}\left(\xi \right)\right)}^{2}\right)}^{2}}\right)}:\xi \in \Psi ;j=1,2,\cdots ,m\right\} \end{array}$$

*Here* $R^{2}$ *is said to be concentration of* R, represented by con(R). *Generally, for all k* ∈ [0, ∞), then

$$\begin{array}{ccc} R^{k} & = & \left\{\frac{\xi }{\left({\left(\mu_{R}^{\left(j\right)}\left(\xi \right)\right)}^{k},\sqrt[{q}]{1-{\left(1-{\left(\nu_{R}^{\left(j\right)}\left(\xi \right)\right)}^{2}\right)}^{k}}\right)}:\xi \in \Psi ;j=1,2,\cdots ,m\right\} \end{array}$$

*The set*

$$\begin{array}{ccc} R^{\frac{1}{2}} & = & \left\{\frac{\xi }{\left(\sqrt{\mu_{R}^{\left(j\right)}\left(\xi \right)},\sqrt[{q}]{1-\sqrt{1-{\left(\nu_{R}^{\left(j\right)}\left(\xi \right)\right)}^{2}}}\right)}:\xi \in \Psi ;j=1,2,\cdots ,m\right\} \end{array}$$

*is known as dilation of* R, *represented as* dil(R).

**Example 3.36** *Suppose that* X={f,g} *is a crisp set, then*

$$R=\{\frac{f}{\left(0.315,0.271),(0.710,0.143),(0.287,0.614\right)},\frac{g}{\left(0.517,0.352),(0.273,0.619),(0.403,0.516\right)}\}$$

*is a qRO3PFS. its matrix form*

$$\begin{array}{c} R=\left[\begin{array}{ccc} \left(0.315,0.271\right) & \left(0.710,0.143\right) & \left(0.287,0.614\right)\\ \left(0.517,0.352\right) & \left(0.273,0.619\right) & \left(0.403,0.516\right) \end{array}\right] \end{array}$$

*We calculate concentration and dilation of R for q* = 2 as

$$\begin{array}{ccc} con(R) & = & \left[\begin{array}{ccc} \left(0.099,0.376\right) & \left(0.504,0.201\right) & \left(0.082,0.782\right)\\ \left(0.267,0.482\right) & \left(0.075,0.787\right) & \left(0.162,0.679\right) \end{array}\right] \end{array}$$

*and*

$$\begin{array}{ccc} dil(R) & = & \left[\begin{array}{ccc} \left(0.561,0.193\right) & \left(0.843,0.101\right) & \left(0.536,0.459\right)\\ \left(0.719,0.253\right) & \left(0.522,0.463\right) & \left(0.635,0.379\right) \end{array}\right] \end{array}$$

**Definition 3.37** *The scalar product of qROmPFN* R *with a scalar α extricated from the crisp set* Ψ *is defined as*

$$\alpha R=\{\frac{\xi }{(\sqrt[{q}]{1-{\left(1-{\left(\mu_{R}^{\left(j\right)}\left(\xi \right)\right)}^{q}\right)}^{\alpha }},{\left(\nu_{R}^{\left(j\right)}\left(\xi \right)\right)}^{\alpha }}):\left(\alpha ⩾0\right),\xi \in \Psi ;j=1,2,\cdots ,m\}$$

**Definition 3.38** *The exponent of qROmPFN* R, *extricated from the crisp set* Ψ *is defined as*

$$R^{\alpha }=\{\frac{\xi }{{(\mu_{R}^{\left(j\right)}\left(\xi \right)}^{\alpha },(\sqrt[{q}]{1-{\left(1-{\left(\nu_{R}^{\left(j\right)}\left(\xi \right)\right)}^{q}\right)}^{\alpha }})}):\left(\alpha ⩾0\right),\xi \in \Psi ;j=1,2,\cdots ,m\}$$

**Definition 3.39** *Let* $R_{1}$ *and* $R_{2}$ *be two qROmPFSs extricated from the classical set* Ψ. *Their cartesian product is represented as under*

$$R_{1}\times R_{2}=\{\frac{\left(\xi_{1},\xi_{2}\right)}{\left(\mu_{R_{1}}^{\left(j\right)}\left(\xi_{1}\right)\mu_{R_{2}}^{\left(j\right)}\left(\xi_{2}\right),\nu_{R_{1}}^{\left(j\right)}\left(\xi_{1}\right)\nu_{R_{2}}^{\left(j\right)}\left(\xi_{2}\right)\right)}:\xi_{1},\xi_{2}\in \Psi ;j=1,2,\cdots ,m\}$$

**Example 3.40** *We consider the two qROmPFSs and Let* Ψ = {*f*, *g*, *h*} *be a crisp set*

$$\begin{array}{ccc} R_{1} & = & \left[\begin{array}{cccc} \left(0.531,0.222\right) & \left(0.412,0.204\right) & \left(0.555,0.301\right) & \left(0.156,0.870\right)\\ \left(0.831,0.231\right) & \left(0.732,0.444\right) & \left(0.830,0.010\right) & \left(0.812,0.110\right)\\ \left(0.766,0.244\right) & \left(0.456,0.140\right) & \left(0.571,0.473\right) & \left(0.611,0.142\right) \end{array}\right]\\ R_{2} & = & \left[\begin{array}{cccc} \left(0.514,0.345\right) & \left(0.819,0.009\right) & \left(0.700,0.227\right) & \left(0.153,0.625\right)\\ \left(0.712,0.106\right) & \left(0.513,0.300\right) & \left(0.729,0.115\right) & \left(0.822,0.200\right)\\ \left(0.632,0.301\right) & \left(1.000,0.000\right) & \left(0.768,0.072\right) & \left(0.000,1.000\right) \end{array}\right] \end{array}$$

*then*

$$\begin{array}{ccc} R_{1}\times R_{2} & = & \left[\begin{array}{cccc} \left(0.273,0.077\right) & \left(0.337,0.002\right) & \left(0.389,0.068\right) & \left(0.024,0.544\right)\\ \left(0.378,0.024\right) & \left(0.211,0.061\right) & \left(0.405,0.035\right) & \left(0.128,0.174\right)\\ \left(0.336,0.067\right) & \left(0.412,0.000\right) & \left(0.426,0.021\right) & \left(0.000,0.870\right)\\ \left(0.427,0.080\right) & \left(0.600,0.004\right) & \left(0.581,0.002\right) & \left(0.124,0.069\right)\\ \left(0.592,0.025\right) & \left(0.376,0.133\right) & \left(0.605,0.001\right) & \left(0.668,0.022\right)\\ \left(0.525,0.070\right) & \left(0.732,0.000\right) & \left(0.637,0.001\right) & \left(0.000,0.110\right)\\ \left(0.394,0.084\right) & \left(0.374,0.001\right) & \left(0.400,0.107\right) & \left(0.094,0.089\right)\\ \left(0.545,0.026\right) & \left(0.234,0.042\right) & \left(0.416,0.054\right) & \left(0.502,0.028\right)\\ \left(0.484,0.073\right) & \left(0.456,0.000\right) & \left(0.439,0.034\right) & \left(0.000,0.142\right) \end{array}\right] \end{array}$$

*In this example, the first row of the matrix defines* $R_{1}\times R_{2}\left(f,f\right)$, *the second row* $R_{1}\times R_{2}\left(f,g\right)$, *the third row* $R_{1}\times R_{2}\left(f,h\right)$ *and so on. We can represent* $R_{1}\times R_{2}\left(g,f\right)$ *rather* $R_{1}\times R_{2}\left(f,g\right)$. *As a result, first row and fourth row are being interchanged, so the result we get does not show its uniqueness*.

**Definition 3.41** *If there exist a one-one correspondence between* $R_{1}$ *and* $R_{2}$, *both taken from the same universal set* Ψ, *are said to be equivalent sets, written as* $R_{1}∼R_{2}$. *This may also be expressed in another way*,

$R_{1}∼R_{2}$ *if and only if* $supp\left(R_{1}\right)=supp\left(R_{2}\right)$.

### 3.2 *α*-cut and (*α*, *β*)-cut of a qROmPFS

This section is based upon *α*-cut and (*α*, *β*)-cut for a qROmPFS, with its certain properties also.

**Definition 3.42** *The α-cut of a qROmPFS* R *may be represented by*

$$\alpha_{R}=\{\xi \in \Psi :\mu_{R}^{\left(j\right)}\left(\xi \right)\geq \alpha ,\forall j=1,2,\cdots ,m\}$$

*It becomes a strong α-cut if*

$$\alpha_{R}^{*}=\{\xi \in \Psi :\mu_{R}^{\left(j\right)}\left(\xi \right)>\alpha ,\forall i=1,2,\cdots ,m\}$$

*Where α* ∈ [0, 1].

**Example 3.43** *Let* R *be a qROmPFS and* Ψ = {*d*, *e*, *f*, *g*, *h*}.

$$\begin{array}{ccc} R & = & \left[\begin{array}{cccc} \left(0.520,0.319\right) & \left(0.618,0.259\right) & \left(0.539,0.171\right) & \left(0.624,0.711\right)\\ \left(0.501,0.243\right) & \left(0.126,0.211\right) & \left(0.678,0.147\right) & \left(0.014,0.537\right)\\ \left(0.668,0.029\right) & \left(0.507,0.433\right) & \left(0.519,0.342\right) & \left(0.971,0.132\right)\\ \left(0.613,0.258\right) & \left(0.559,0.218\right) & \left(0.844,0.257\right) & \left(0.522,0.164\right)\\ \left(0.291,0.160\right) & \left(0.343,0.184\right) & \left(0.435,0.855\right) & \left(0.332,0.974\right) \end{array}\right] \end{array}$$

*then* 0.520-*cut of* R *is*

$$0.520_{R}=\left\{d,f,g\right\}$$

*and strong* 0.520-*cut of* R *is*

$$0.520_{R}^{*}=\left\{f,g\right\}$$

**Proposition 3.44** *Let* R *be a qROmPFS defined over* Ψ, *then*

- $0_{R}=X$, *which is universe*.
- $1_{R}^{*}=\phi$, *which is null set*.

**Proposition 3.45** *Let* $R_{1}$ *and* $R_{2}$ *be qROmPFSs over the univese* Ψ *and α*, *β* ∈ [0, 1], *then we have the following*

- $\alpha_{R_{1}}^{*}⊑\alpha_{R_{1}}$.
- $\alpha_{R_{1}⊔R_{2}}=\alpha_{R_{1}}⊔\alpha_{R_{2}}$.
- $\alpha_{R_{1}⊓R_{2}}=\alpha_{R_{1}}⊓\alpha_{R_{2}}$.
- $\alpha_{R_{1}⊔R_{2}}^{*}=\alpha_{R_{1}}^{*}⊔\alpha_{R_{2}}^{*}$.
- $\alpha_{R_{1}⊓R_{2}}^{*}=\alpha_{R_{1}}^{*}⊓\alpha_{R_{2}}^{*}$.
- If *α* ≥ *β*, then $\alpha_{R_{1}}⊑\beta_{R_{1}}$.

**Proof 6**

- *Suppose that* $\xi \in \alpha_{R_{1}}^{*}$,but $\mu_{R_{1}}^{\left(j\right)}>\alpha$ *for all permissible viewpoint of j*. *It means that* $\mu_{R_{1}}^{\left(j\right)}\geq \alpha$ *for every permissible viewpoint of j, proved*.
- *For all permissible values of j, Let*
  $$\begin{array}{ccc} \xi & \in & \alpha_{R_{1}⊔R_{2}}\\ \Leftrightarrow \alpha_{R_{1}⊔R_{2}}\left(\xi \right) & \geq & \alpha \\ \Leftrightarrow \max\{\alpha_{R_{1}}\left(\xi \right),\alpha_{R_{2}}\left(\xi \right)\} & \geq & \alpha \\ \Leftrightarrow \alpha_{R_{1}}\left(\xi \right)\geq \alpha & or & \alpha_{R_{2}}\left(\xi \right)\geq \alpha \\ \Leftrightarrow \xi \in \alpha_{R_{1}} & or & \xi \in \alpha_{R_{2}}\\ \Leftrightarrow \xi & \in & \alpha_{R_{1}}⊔\alpha_{R_{1}} \end{array}$$
- *For all permissible values of j, we suppose that*
  $$\begin{array}{ccc} \xi & \in & \alpha_{R_{1}⊓R_{2}}\\ \Leftrightarrow \alpha_{R_{1}⊓R_{2}}\left(\xi \right) & \geq & \alpha \\ \Leftrightarrow \min\{\alpha_{R_{1}}\left(\xi \right),\alpha_{R_{2}}\left(\xi \right)\} & \geq & \alpha \\ \Leftrightarrow \alpha_{R_{1}}\left(\xi \right)\geq \alpha & and & \alpha_{R_{2}}\left(\xi \right)\geq \alpha \\ \Leftrightarrow \xi \in \alpha_{R_{1}} & and & \xi \in \alpha_{R_{2}}\\ \Leftrightarrow \xi & \in & \alpha_{R_{1}}⊓\alpha_{R_{1}} \end{array}$$
- *can be discussed on the same pattern of (ii), the only difference is that instead of* ≥, *we can use* >.
- *can be discussed on the same pattern of (iii), the only difference is that instead of* ≥, *we can use* >.
- *For all permissible values of j, we consider that*
  $$\begin{array}{ccc} \xi & \in & \alpha_{R_{1}}\\ \Rightarrow \alpha_{R_{1}}\left(\xi \right) & \geq & \alpha \geq \beta \\ \Rightarrow \alpha_{R_{1}}\left(\xi \right) & \geq & \beta \\ \Rightarrow \xi & \in & \beta_{R_{1}}\\ \Rightarrow \alpha_{R_{1}} & ⊑ & \beta_{R_{1}} \end{array}$$

**Example 3.46** *Let* R *be a qROmPFS and* Ψ = {*d*, *e*, *f*, *g*, *h*}.

$$\begin{array}{ccc} R & = & \left[\begin{array}{cccc} \left(0.520,0.319\right) & \left(0.618,0.259\right) & \left(0.539,0.171\right) & \left(0.624,0.711\right)\\ \left(0.501,0.243\right) & \left(0.126,0.211\right) & \left(0.678,0.147\right) & \left(0.014,0.537\right)\\ \left(0.668,0.029\right) & \left(0.507,0.433\right) & \left(0.519,0.342\right) & \left(0.971,0.132\right)\\ \left(0.613,0.258\right) & \left(0.559,0.218\right) & \left(0.844,0.257\right) & \left(0.522,0.164\right)\\ \left(0.291,0.160\right) & \left(0.343,0.184\right) & \left(0.435,0.855\right) & \left(0.332,0.974\right) \end{array}\right] \end{array}$$

*If we take α* = 0.291, *then we have*

$$\begin{array}{ccc} \alpha_{R} & = & \left\{d,e,f,g,\right\}\\ \Rightarrow \alpha_{R}^{c} & = & \left\{h\right\} \end{array}$$

*and*

$$\begin{array}{ccc} {\left(1-\alpha \right)}_{R}^{*} & = & \varphi \\ ∴{\left({\left(1-\alpha \right)}_{R}^{*}\right)}^{c} & = & X. \end{array}$$

**Proposition 3.47** *Let* R *be a qROmPFS over* Ψ, *then contrary to fuzzy sets*

$$\alpha_{R}^{c}\neq {\left({\left(1-\alpha \right)}_{R}^{*}\right)}^{c}$$

**Definition 3.48** *If we consider a classical set for* 0 ≤ *α* ≤ 1 *designating various α*-*cuts for a particular qROmPFS* R *is called a level set of* R, *we can nominate it with* Λ(R) *i.e*.

$$\Lambda \left(R\right)=\{\alpha \in \left[0,1\right]:\mu_{R}^{\left(j\right)}\left(\xi \right)=\alpha ,\forall j=1,2,\cdots ,m\}$$

**Proposition 3.49** *If* $R_{1}$ *and* $R_{2}$ *are two qROmPFSs over* Ψ, *then* $R_{1}⊑R_{2}$ *if and only if* $\alpha_{R_{1}}⊑\alpha_{R_{2}}$, *for all α* ∈ [0, 1].

**Proof 7** *We consider that* $\alpha_{R_{1}}⊑\alpha_{R_{2}}$ *assuming that* $R_{1}⊑R_{2}$. *To prove this we take it on the contrary basis. Let there exists an α* ∈ [0, 1] *such that* $\alpha_{R_{1}}⊈\alpha_{R_{2}}$. *So, we must have minimum one* $\xi \in \alpha_{R_{1}}$ *that must not be in* $\alpha_{R_{2}}$. *Then, we may define* $\mu_{R_{1}}^{\left(j\right)}\left(\xi \right)\geq \alpha$, $\mu_{R_{2}}^{\left(j\right)}\left(\xi \right)≱\alpha$. *On the other hand*, $\mu_{R_{2}}^{\left(j\right)}\left(\xi \right)<\alpha$. *Now by combining both inequalities we have* $\mu_{R_{1}}^{\left(j\right)}\left(\xi \right)\geq \alpha >\mu_{R_{2}}^{\left(j\right)}\left(\xi \right)$ i.e. $\mu_{R_{1}}^{\left(j\right)}\left(\xi \right)>\mu_{R_{2}}^{\left(j\right)}\left(\xi \right)$ *which is a contradiction*.

*Conversely, suppose that* $\alpha_{R_{1}}⊑\alpha_{R_{2}}$. *We consider that* $R_{1}⊑R_{2}$ *i.e*. $\mu_{R_{1}}^{\left(j\right)}\left(\xi \right)\leq \mu_{R_{2}}^{\left(j\right)}\left(\xi \right)$ and $\nu_{R_{1}}^{\left(j\right)}\left(\xi \right)\geq \nu_{R_{2}}^{\left(j\right)}\left(\xi \right)$, *for all* $\xi \in R_{1}$. *Contradictory we Suppose* $\xi^{\prime }\in R_{1}$ *as follows* $\mu_{R_{1}}^{\left(j\right)}\left(\xi^{\prime }\right)≰\mu_{R_{2}}^{\left(j\right)}\left(\xi^{\prime }\right)$ *i.e*. $\mu_{R_{1}}^{\left(j\right)}\left(\xi^{\prime }\right)>\mu_{R_{2}}^{\left(j\right)}\left(\xi^{\prime }\right)$. *This symbolizes* $\xi^{\prime }\in \alpha_{R_{1}}$ *yet* $\xi^{\prime }\notin \alpha_{R_{2}}$, *this completes the proof*.

**Corollary 3.50** *Let* $R_{1}$ *and* $R_{2}$ *be qROmPFSs over* Ψ, *then for all* *α* ∈ [0, 1]

- $R_{1}⊑R_{2}$ if and only if $\alpha_{R_{1}}^{*}⊑\alpha_{R_{2}}^{*}$.
- $R_{1}=R_{2}$ if and only if $\alpha_{R_{1}}=\alpha_{R_{2}}$.

**Definition 3.51** *The* (*α*, *β*)-*cut for a qROmPFS* R, *represented by*

$${\left(\alpha ,\beta \right)}_{R}=\{\xi \in \Psi :\mu_{R}^{\left(j\right)}\left(\xi \right)\geq \alpha \;and\;\nu_{R}^{\left(j\right)}\left(\xi \right)\leq \beta ,\forall j=1,2,\cdots ,m\}$$

*for* 0 ≤ *α*, *β* ≤ 1 *and* *α* + *β* ∈ [0, 1].

**Example 3.52** *From qROmPFS the* R *shown in Example 3.46*, (0.520, 0.319)-*cut as*

$${\left(0.520,0.319\right)}_{R}=\left\{f,g\right\}$$

## 4 MCDM for robotic agri-farming

This section gives multi criteria decision making (MCDM) to rank the alternative from high significance to low significance. In MCDM the decision makers (DMs) select the alternatives by themselves, to choose the best alternative from a set of suitable alternatives under a particular situation. Although there exist numerous aggregation methods, in the current context we propose extension of grey relational analysis (GRA) towards qROmPFSs for MCDM. An application we are discussing here as a reference is related to farming. For the optimal solution the alternatives are collated against the selected criteria. So we can say that MCDM is a composition of set of alternatives, set of multiple criteria and their comparison. We have to choose those alternatives with the help of MCDM which suits in all the way for best solution.

**Case study**

Farming is an activity or business of growing crops and raising livestock. Farming involves rearing animals and growing crops, that give us food and raw materials also. Farming began almost thousands of years ago but we cannot tell the exact time and origin. Farming is not just a job it’s a way of life. It also gave a rise to the human civilization and without it our survival on earth is impossible. Once the American president George Washington gave an admirable statement about the agriculture “agriculture is the most helpful, most useful and most noble employment of men”. Actually we all are farmers as everyone of us love gardening either at home or in the fields. At home we grow plants in small mud pots while in field we are free to grow plants, trees, or crops. This habit of love towards gardening must be evergreen either you are young or old. Now a days in the name of development, industrialization and housing societies, we are demolishing our homeland and minimizing the cultivating lands. In this process of land destroying the food prices will shoot up and we have to pay much more for our daily requirement of food items. Agriculture is the science and art of cultivating plants and increasing livestock. As a whole if we discuss there are almost top 10 types (arable farming, pastoral farming, mixed farming, subsistence farming, commercial farming, extensive farming,intensive farming, nomadic farming, sedentary farming, poultry farming, fish farming) of farming practised through out the world.

As the world population is rising fast, people need more food for their survival. Due to this high demand of food, the farmers are also facing much pressure to enhance the crop production. To overcome this situation farmers must focus to improve the yield production by using agricultural robots. Creativity beyond the innovation is the use of robots in the field of agriculture. The agricultural farming works like an industry and in the present era it is going to become a high-tech industry. As the technology is advancing so fast, the agricultural capacities of farmers are also increasing rapidly. The robotics and automation technology is now boosting up the production yields. Robotic applications in the field of agriculture are (harvesting, weeding, pruning, seeding, spraying, sorting and packing etc.). The agriculture robots are also known as “agribots” or “agri-robots”. In future the agribots will perform a major role in the field of agriculture. We are considering here only one application, use of robots in Horticulture. The agriculture of food plants, material plants, comfort plants and decorative plants is called horticulture. A new generation robot named as “Terra Sentia” (the smallest robot having a width 12.5 inches and hight 12.5 inches almost with a weight of 30 pounds) looks like a lawn mover with each side furnished with high-resolution cameras, navigates a field by emitting laser pulses to scan it. To portrait the field, health and size of plants, stem diameter, counting of plants, fruit producing plants. It can also be used for the plant breeding scientifically. This robot is effectively proven in a wide range of fields like cotton, corn, wheat, soybean, strawberries, tomatoes, apple orchards, citrus crops, sorghum, vineyards and almond farms.

We are analyzing here the efficiency of the robots in farming. The attributes of robotic agri-farming are given below.

- Automating manual tasks: By utilizing the automation farmers improve their efficiency by spending less time on tasks,more time on the improvement.
- High quality production: The quality products are influenced by certain factors in farming i.e (soil,time of ripeness, climate, fertilizer etc). Maturity level anddegree of dryness matter in the yield of cereals (wheat, barley,rice,oats etc.)
- Reducing the need for manual labor: As the cost of labor is much high in the field of agriculture i.e. (paying to skilled worker and manual labor).
- Lowering production cost: There is an advanced way for lowering the production cost by using robots in the field of agriculture. We have to manage some uncontrollable factors those minimize the profit margin, weather conditions,purchasing different brands of seeds,utilizing access amount of chemicals.
- Completion of a complicated task: The scientists,technologists,researchers and farmers all are agreed that the use of automation will complete the complicated task in a simple and easy way.
- Consistent role to fulfill a task: For consistent role, the farm must be run under the artificial intelligence (automate the entire process of farming) from seeding to harvesting.
- Perfection and accuracy in placement: The placement of plants is very much important in the field. The accuracy will create perfection. Nursing operation automation concludes propagation, grafting, and spacing.

### 4.1 Grey relational analysis with qROmPFSs

First we apply an optimization technique “grey relational analysis” (GRA) to seek the optimal alternative using the notion of compromise solution (the solution which is closest to the ideal solution and farthest from negative ideal solution is acknowledged as compromise solution). We study how qROmPFSs may be utilized in MCDM by using GRA. Initially we extend GRA to qROmPFSs and observing its results with a different approach “Choice Value Method” (CVM) to analyze it. To make it clear we are discussing same application for both methods.

To reach the maximum crop potential, it’s very much important to get the crop production properly from it’s sowing stage. Moreover we have to use MCDM to verify the crop which is more profitable for the former. Although the outputs are based upon inputs (money, labor, soil, climate, fertilizer etc). The lingual phrases for concluding alternatives are given in Table 2.

**Table 2:** Lingual phrases for concluding alternatives.

Linguistic Terms	Fuzzy Weights
Less crop production (LP)	[0.000, 0.300]
Ordinary crop production (OP)	(0.300, 0.500]
Good crop production (GP)	(0.500, 0.700]
More crop production (MP)	(0.700, 0.900]
Exceptional crop production (EP)	(0.900, 1.000]

We may set up a technique by discussing each and every step, as under:

**Algorithm-1 (Grey relational analysis)**

Step 1: Understand the problem and what to do: Consider that *V* = {*ξ*_*j*_: *j* = 1, 2, ⋯, *n*} is a set (with restrictions) of alternatives under discussion and *D* = {*d*_*k*_: *k* = 1, 2, ⋯, *m*} be a group of decision makers (DMs). Consider a set of attributes or criterion. Then the (*j*, *k*)^*th*^ entry of the qROmPFS matrix illustrate the weight proposed by *k*^*th*^ DM to *j*^*th*^ alternative.

Step 2: Compute matrix of weighted parameters as $A={\left[w_{jk}\right]}_{n\times m}$, where *w*_*jk*_ shows fuzzy weights allocated by the decision makers *d*_*j*_ to the attributes *α*_*j*_ by allotting lingual values given in Table 2.

Step 3: Compute the normalized matrix $\dot{A}={\left[{\dot{w}}_{jk}\right]}_{n\times m}$ by using ${\dot{w}}_{jk}=\frac{w_{jk}}{\sqrt{\Sigma_{j=1}^{n}w_{jk}^{2}}}$. Then compute the weight vector of DMs $W=(w_{1},w_{2},\cdots ,w_{m})$, where $w_{j}=\frac{\sum_{j=1}^{n}{\dot{w}}_{jk}}{\sum_{k=1}^{m}\sum_{j=1}^{n}{\dot{w}}_{jk}}$.

Step 4: Construct qROmPFS *κ*_*D*_ then compute the matrix $S_{D}$ by replacing the q-ROFNs $\left(\mu_{R},\nu_{R}\right)$ with their corresponding score function using $s={\left(\mu_{R}\right)}^{q}-{\left(\nu_{R}\right)}^{q}$ (*q* > 1).

Step 5: Compute the highest value *h*_*j*_ and the least value *l*_*j*_ of the matrix $S_{D}$. Then there are two options:

- least-the-better (for non-beneficial objects) by using the formula
  $$\begin{array}{c} \lceil^{-}\left(j\right)=\frac{h_{j}-d_{j}}{h_{j}-l_{j}}\in \left[0,1\right] \end{array}$$ (1)
- highest-the-better (for beneficial objects) by using the formula
  $$\begin{array}{c} \lceil^{+}\left(j\right)=\frac{d_{j}-l_{j}}{h_{j}-l_{j}}\in \left[0,1\right] \end{array}$$ (2)

We construct qROmPFS decision matrix $B={\left[\xi_{ik}\right]}_{n\times m}$ by using either least-the-better or highest-the-better formulas.

Step 6: Calculating the grey relational coefficient (GRC) by using the formulae

$$\begin{array}{c} GRC=\frac{1}{\Sigma_{k=1}^{m}w_{k}\left|\xi_{ik}-1\right|+1},\left(i=1,\cdots ,n\right) \end{array}$$ (3)

Step 7: Now rank the alternatives according to the (GRC) values. The crop with the highest GRC is the most valuable growing crop.

We are going to use the proposed GRA based algorithm for real life application with available data for farming purpose.

**Example 4.1** *A farmer running a big agriculture farm, it may be a costly venture, but he want to earn a big revenue from his farm. He belong to a farming family, inherit skills and passion to do full time sustainable agri-farming. He aims to live a happy life and excellent education facilities for his kids. For the purpose of earning high-profit, he want to upgrade his vision through robotics, that might fulfil his ideas, goals and concerns by minimizing available resources and making this profession a high-tech profession. To make it a moneymaking business, the farmer gave this task to his sons to have a unanimous decision based upon the scientifically managed approach*.

*Step 1: Identify the problem*: *Let* *V* = {*ξ*_*j*_: *j* = 1, 2, ⋯, 7} *be the set of alternatives for robotic agri-farming, and* $D=\{d_{j}:j=1,2,3,4\}$ *be the family of DMs. We consider the set of attributes or criterion for robotic agri-farming as given below*.
  $$\begin{array}{ccc} \alpha_{1} & = & Automatingmanualtasks,\\ \alpha_{2} & = & Highqualityproduction,\\ \alpha_{3} & = & Reducingtheneedformanuallabor,\\ \alpha_{4} & = & Loweringproductioncost,\\ \alpha_{5} & = & Completionofacomplicatedtask,\\ \alpha_{6} & = & Consistentroletofulfillatask,\\ \alpha_{7} & = & Perfectionandaccuracyinplacement. \end{array}$$
*Step 2: Here weighted parameter matrix is*
  $$\begin{array}{ccc} A & = & {\left[w_{ij}\right]}_{7\times 4}\\ & = & \left[\begin{array}{cccc} MP & EP & GP & OP\\ GP & EP & MP & EP\\ EP & LP & OP & MP\\ GP & EP & MP & LP\\ EP & MP & EP & OP\\ LP & EP & OP & GP\\ OP & MP & EP & GP \end{array}\right]\\ & = & \left[\begin{array}{cccc} 0.780 & 0.915 & 0.575 & 0.358\\ 0.575 & 0.915 & 0.780 & 0.915\\ 0.915 & 0.120 & 0.358 & 0.780\\ 0.575 & 0.915 & 0.780 & 0.120\\ 0.915 & 0.780 & 0.915 & 0.358\\ 0.120 & 0.915 & 0.358 & 0.575\\ 0.358 & 0.780 & 0.915 & 0.575 \end{array}\right] \end{array}$$
  *where* *w*_*ij*_ *shows the weight assigned to the attribute* *α*_*i*_ *by the decision maker* *d*_*j*_ *by using the lingual values given in* Table 2.
*Step 3: Matrix with normalized weights*:
  $$\begin{array}{ccc} \dot{A} & = & {\left[{\dot{w}}_{ij}\right]}_{7\times 4}\\ & = & \left[\begin{array}{cccc} 0.444 & 0.428 & 0.308 & 0.232\\ 0.327 & 0.428 & 0.418 & 0.593\\ 0.521 & 0.056 & 0.192 & 0.506\\ 0.327 & 0.428 & 0.418 & 0.078\\ 0.521 & 0.364 & 0.491 & 0.232\\ 0.068 & 0.428 & 0.192 & 0.373\\ 0.204 & 0.364 & 0.491 & 0.373 \end{array}\right] \end{array}$$
  *which gives the weight vector* *W* = (0.246, 0.255, 0.256, 0.243).
*Step 4: Construct qROmPFS* *κ*_*D*_ *as shown in* Table 3. *Construct matrix* $S_{D}$ *by replacing q-ROFNs* $\left(\mu_{R},\nu_{R}\right)$ *by their score function using* $s={\left(\mu_{R}\right)}^{q}-{\left(\nu_{R}\right)}^{q}$, *for* *q* = 4, *we obtain the following matrix*
  $$\begin{array}{c} S_{D}=[\begin{array}{cccc} 0.608 & 0.967 & 1.000 & 0.000\\ 0.493 & 0.729 & 0.769 & 0.675\\ 0.656 & 0.512 & 0.655 & 0.344\\ 0.699 & 0.741 & 0.476 & 0.890\\ 0.478 & 0.408 & 0.531 & 0.128\\ 0.418 & 0.097 & 0.388 & 0.305\\ 0.310 & 0.313 & 0.320 & 0.262 \end{array}] \end{array}$$
*Step 5: Constructing qROmPFS decision matrix by using “highest is the better” as given in* Eq (2).
  $$\begin{array}{ccc} B & = & \left[\begin{array}{cccc} 0.568 & 0.982 & 1.000 & 0.000\\ 0.564 & 0.805 & 0.825 & 0.691\\ 0.696 & 0.549 & 0.696 & 0.409\\ 0.732 & 0.812 & 0.550 & 0.952\\ 0.583 & 0.381 & 0.540 & 0.250\\ 0.405 & 0.234 & 0.051 & 0.519\\ 0.420 & 0.275 & 0.497 & 0.199 \end{array}\right] \end{array}$$
*Step 6: Now calculating the gray relational coefficient (GRC) by using formula as given in* Eq (3). *The values of GRC are given in* Table 4.
*Step 7: The preference order, with “highest-the-better”, of the alternatives is*
  $$\xi_{4}≻\xi_{2}≻\xi_{1}≻\xi_{3}≻\xi_{5}≻\xi_{6}=\xi_{7}$$

**Fig 1:**
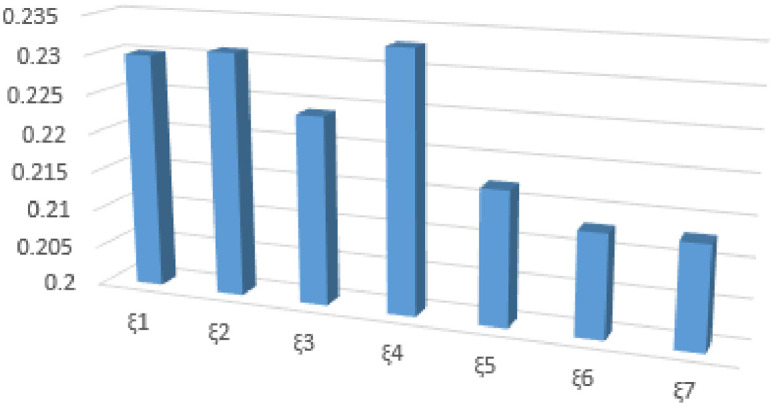
Rank of alternatives in bar graph.

*The above ranking of (GRC) is represented in* Fig 1. *From the (GRC) ranking, The farmer is in a better position of earning more profit When he reduces the production cost. On the other hand with the help of robotics he is in a position to fulfill a complicated task more easily*.

**Table 3:** qROmPFS.

*κ*_*D*_	*d*_1_	*d*_2_	*d*_3_	*d*_4_
*ξ*_1_	(0.573,0.326)	(0.870,0.334)	(0.883,0.262)	(0.165,0.911)
*ξ*_2_	(0.570,0.630)	(0.743,0.477)	(0.757,0.391)	(0.661,0.288)
*ξ*_3_	(0.665,0.438)	(0.559,0.594)	(0.665,0.440)	(0.459,0.733)
*ξ*_4_	(0.691,0.346)	(0.748,0.460)	(0.560,0.643)	(0.849,0.491)
*ξ*_5_	(0.584,0.658)	(0.439,0.667)	(0.553,0.558)	(0.345,0.856)
*ξ*_6_	(0.456,0.661)	(0.333,0.871)	(0.202,0.659)	(0.538,0.784)
*ξ*_7_	(0.467,0.761)	(0.363,0.741)	(0.522,0.769)	(0.308,0.774)

**Table 4:** GRC of alternatives.

Alternative (*ξ*_*j*_)	GRC
*ξ*_1_	0.230
*ξ*_2_	0.231
*ξ*_3_	0.224
*ξ*_4_	0.233
*ξ*_5_	0.217
*ξ*_6_	0.213
*ξ*_7_	0.213

### 4.2 Generalized choice value method

Decision making is a dynamic part of business, economics, social sciences and real world problems. It marks out from daily low level operational assessments at low-ranking management level to long-term strategic planning faced by senior administration. Conclusions that are produced at any level can cause serious or bad consequences, but is there an explicit layout that decision makers should adopt in order to assure success, or should override the regular plan of attack?

The decision makers should hire many factors into account before reaching a unanimous decision. So it is essential to ascertain all those components taken before the determination, must be finalized. In parliamentary law it is essential that all the indispensable facts and figures should be scrutinized before implementation. It is indispensable to coordinate the decision making with a taxonomic attitude.

Mathematics provides a wide range of algorithms to compare and assist in reaching conclusions on scientific evidence. In this fragment, we render the Algorithm 2 which is the extended form of well-known choice value method (CVM) named as generalized choice value method (GCVM) for MCDM under qROmPFSs.

**Algorithm 2 (Generalized choice value method)**

**Step 1**: Understand the problem and what to do: Consider that *V* = {*ξ*_*j*_ : *j* = 1, 2, ⋯, *n*} is a set (with restrictions) of alternatives under discussion and *D* = {*d*_*k*_: *k* = 1, 2, ⋯, *m*} be a group of decision makers (DMs). Consider a set of attributes or criterion. Then the (*j*, *k*)^*th*^ entry of the qROmPFS matrix illustrate the weight proposed by *k*^*th*^ DM to *j*^*th*^ alternative.

**Step 2**: Compute matrix of weighted parameters as $A={\left[w_{jk}\right]}_{n\times m}$, where *w*_*jk*_ shows fuzzy weights allocated by the decision makers *d*_*j*_ to the attributes *α*_*j*_ given in Table 2.

**Step 3**: Compute the normalized matrix $\dot{A}={\left[{\dot{w}}_{jk}\right]}_{n\times m}$ by using ${\dot{w}}_{jk}=\frac{w_{jk}}{\sqrt{\Sigma_{j=1}^{n}w_{jk}^{2}}}$.

Then compute the weight vector $W=(w_{1},w_{2},\cdots ,w_{m})$, where $w_{j}=\frac{\sum_{j=1}^{n}{\dot{w}}_{jk}}{\sum_{k=1}^{m}\sum_{j=1}^{n}{\dot{w}}_{jk}}$.

**Step 4**: Construct qROmPFS *κ*_*D*_.

**Step 5**: Find the matrix of choice values using $C=\frac{1}{\Sigma W(e_{i})}(\kappa_{D}\times W^{t})$.

**Step 6**: Compute the value of score function *s* for each $\varrho_{i}$ using $s={\left(\mu_{R}\right)}^{q}-{\left(\nu_{R}\right)}^{q}$ (*q* > 1).

In case of a tie, compute the values of accuracy function $a(\varrho_{i})$ using $a={\left(\mu_{R}\right)}^{q}+{\left(\nu_{R}\right)}^{q}$ (*q* > 1).

**Step 7**: The $\varrho_{i}$ for which $s(\varrho_{i})$ is maximum is the desired alternative.

**Example 4.2** Now we use the data collected in Example 4.1 and apply Algorithm 2. We use GCVM as given by Algorithm 2 for the selection of optimal alternative among a list of feasible alternative. Lastly, we give a comparison of the ranking computed by GRA and GCVM.

The first four steps of Algorithm 1 and Algorim 2 are same. So we proceed with step 5 as follows.

Consider the qROmPF-matrix

$$\begin{array}{c} \kappa_{D}=[\begin{array}{cccc} \left(0.573,0.326\right) & \left(0.870,0.334\right) & \left(0.883,0.262\right) & \left(0.165,0.911\right)\\ \left(0.570,0.630\right) & \left(0.743,0.477\right) & \left(0.757,0.391\right) & \left(0.661,0.288\right)\\ \left(0.665,0.438\right) & \left(0.559,0.594\right) & \left(0.665,0.440\right) & \left(0.459,0.733\right)\\ \left(0.691,0.346\right) & \left(0.748,0.460\right) & \left(0.560,0.643\right) & \left(0.849,0.491\right)\\ \left(0.584,0.658\right) & \left(0.439,0.667\right) & \left(0.553,0.558\right) & \left(0.345,0.856\right)\\ \left(0.456,0.661\right) & \left(0.333,0.871\right) & \left(0.202,0.659\right) & \left(0.538,0.784\right)\\ \left(0.467,0.761\right) & \left(0.363,0.741\right) & \left(0.522,0.769\right) & \left(0.308,0.774\right) \end{array}] \end{array}$$

and the weight vector *W* = (0.246, 0.255, 0.256, 0.243).

Then the matrix C of choice values for qROFS-matrix is given by

$$\begin{array}{ccc} C & = & \frac{1}{\Sigma W_{ij}}(\kappa_{D}\times W^{t})\\ & = & \frac{1}{1.000}[\begin{array}{cccc} \left(0.573,0.326\right) & \left(0.870,0.334\right) & \left(0.883,0.262\right) & \left(0.165,0.911\right)\\ \left(0.570,0.630\right) & \left(0.743,0.477\right) & \left(0.757,0.391\right) & \left(0.661,0.288\right)\\ \left(0.665,0.438\right) & \left(0.559,0.594\right) & \left(0.665,0.440\right) & \left(0.459,0.733\right)\\ \left(0.691,0.346\right) & \left(0.748,0.460\right) & \left(0.560,0.643\right) & \left(0.849,0.491\right)\\ \left(0.584,0.658\right) & \left(0.439,0.667\right) & \left(0.553,0.558\right) & \left(0.345,0.856\right)\\ \left(0.456,0.661\right) & \left(0.333,0.871\right) & \left(0.202,0.659\right) & \left(0.538,0.784\right)\\ \left(0.467,0.761\right) & \left(0.363,0.741\right) & \left(0.522,0.769\right) & \left(0.308,0.774\right) \end{array}][\begin{array}{c} 0.246\\ 0.255\\ 0.256\\ 0.243 \end{array}]\\ & = & \left[\begin{array}{c} \left(0.6290,0.4538\right)\\ \left(0.6841,0.4467\right)\\ \left(0.5879,0.5500\right)\\ \left(0.7104,0.4863\right)\\ \left(0.4810,0.6828\right)\\ \left(0.3795,0.7439\right)\\ \left(0.4159,0.7611\right) \end{array}\right] \end{array}$$

The values of the score function for *q* = 5 are as demonstrated in Table 5. Table 5 demonstrates that

$$\xi_{4}≻\xi_{2}≻\xi_{1}≻\xi_{3}≻\xi_{5}≻\xi_{6}≻\xi_{7}$$

**Table 5:** Score values for alternatives with *q* = 5.

*X*	*S* = *μ*^5^ − *ν*^5^	Ranking
*ξ*_1_	0.0792	3
*ξ*_2_	0.1320	2
*ξ*_3_	0.0199	4
*ξ*_4_	0.1539	1
*ξ*_5_	−0.1227	5
*ξ*_5_	−0.2199	6
*ξ*_5_	−0.2429	7

For *q* = 6 are as demonstrated in Table 6. Table 6 demonstrates that

$$\xi_{4}≻\xi_{2}≻\xi_{1}≻\xi_{3}≻\xi_{5}≻\xi_{6}≻\xi_{7}$$

**Table 6:** Score values for alternatives with *q* = 6.

*X*	*S* = *μ*^6^ − *ν*^6^	Ranking
*ξ*_1_	0.0532	3
*ξ*_2_	0.0946	2
*ξ*_3_	0.0136	4
*ξ*_4_	0.1153	1
*ξ*_5_	−0.0890	5
*ξ*_5_	−0.1665	6
*ξ*_5_	−0.1892	7

For *q* = 7, the score values are as demonstrated in Table 7. Table 7 demonstrates that

$$\xi_{4}≻\xi_{2}≻\xi_{1}≻\xi_{3}≻\xi_{5}≻\xi_{6}≻\xi_{7}$$

**Table 7:** Score values for alternatives with *q* = 7.

*X*	*S* = *μ*^7^ − *ν*^7^	Ranking
*ξ*_1_	0.0350	3
*ξ*_2_	0.0666	2
*ξ*_3_	0.0090	4
*ξ*_4_	0.0849	1
*ξ*_5_	−0.0632	5
*ξ*_5_	−0.1249	6
*ξ*_5_	−0.1458	7

**Fig 2:**
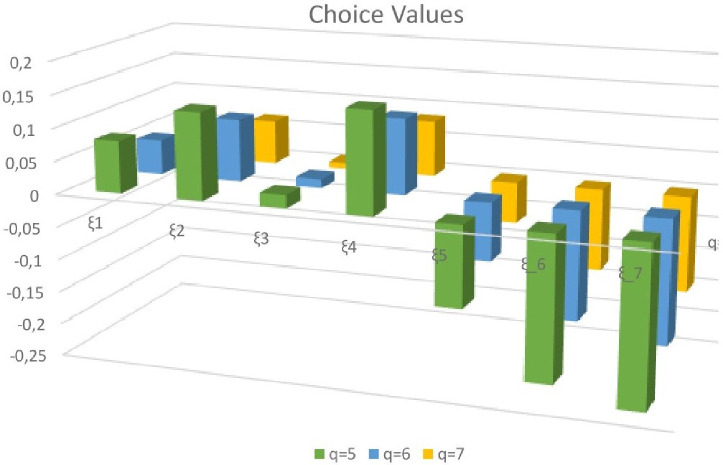
3D bar chart of ranking of alternatives.

From the rank table this is clear that the first preference of selection board will be *ξ*_4_, while their 2nd preference will be *ξ*_2_. The rankings of alternatives for different values of *q* chosen is depicted in Fig 2.

### 4.3 Comparison analysis

We solved the problem of robotic farming by using GRA and GCVM which are described by Algorithm 1 and Algorithm 2. We observe that the optimal solution attained by both techniques is the same which shows the validity of proposed techniques. These approaches are also compared with some other existing methods as indicated in the Table 8 as given below listing the results of the comparison in the final ranking of top seven alternatives.

**Table 8:** Comparison analysis of final ranking with GRA and GCVM.

Method	Ranking of alternatives	Optimal alternative
Algorithm 1 (Proposed)	*ξ*_4_ ≻ *ξ*_2_ ≻ *ξ*_1_ ≻ *ξ*_3_ ≻ *ξ*_5_ ≻ *ξ*_6_ = *ξ*_7_	*ξ*_4_
Algorithm 2 (Proposed)	*ξ*_4_ ≻ *ξ*_2_ ≻ *ξ*_1_ ≻ *ξ*_3_ ≻ *ξ*_5_ ≻ *ξ*_6_ ≻ *ξ*_7_	*ξ*_4_
Algorithm (Eraslan and Karaaslan [29])	*ξ*_4_ ≻ *ξ*_2_ ≻ *ξ*_1_ ≻ *ξ*_3_ ≻ *ξ*_5_ ≻ *ξ*_6_ ≻ *ξ*_7_	*ξ*_4_
Algorithm (Kumar and Garg [30])	*ξ*_4_ ≻ *ξ*_2_ ≻ *ξ*_1_ ≻ *ξ*_3_ ≻ *ξ*_5_ ≻ *ξ*_6_ ≻ *ξ*_7_	*ξ*_4_
Algorithm (Zhang and Xu [34])	*ξ*_4_ ≻ *ξ*_2_ ≻ *ξ*_1_ ≻ *ξ*_3_ ≻ *ξ*_5_ ≻ *ξ*_6_ ≻ *ξ*_7_	*ξ*_4_

The superiority and validity of the proposed approach is shown in the Table 9. The proposed model of qROmPFS is superior to existing models like fuzzy set, IFS, PFS, qROFS, mPFS and PmPFS. For *q* = 2 qROmPFS reduces to PmPFS. For *m* = 1 it reduces to qROFS. For *q* = 2 & *m* = 1 it reduces to PFS. In fact each of the model IFS, PFS, qROFS, mPFS and PmPFS are the special cases of qROmPFS.

**Table 9:** Comparison of q-rung orthopair *m*-polar fuzzy set with existing models.

Set theoretic	Membership	Non-membership	Multi-polarity	Broader
models	grades	grades		space
Fuzzy set [1]	✔	✕	✕	✕
IFS [2]	✔	✔	✕	✕
PFS [4, 5]	✔	✔	✕	✕
qROFS [6]	✔	✔	✕	✔
*m*-polar fuzzy set [37]	✔	✕	✔	✕
PmPFS [14, 17]	✔	✔	✔	✕
qROmPFS (proposed)	✔	✔	✔	✔

## 5 Conclusion

In some real life situations the multi-polarity of membership and non-membership grades become necessary to express vague and uncertain information in a broader space, in order to deal with such situations, the concept of q-rung orthopair m-polar fuzzy set (qROmPFS) is introduced as a new hybrid model of q-rung orthopair fuzzy set and m-polar fuzzy set. We presented some fundamental operations on qROmPFSs along with their necessary results. We extracted crisp sets like support, core and height from qROmPFS. To contrive the conception successfully, we have added several illustrations to explain these concepts. The grey relational analysis (GRA) and generalized choice value method (GCVM) based algorithms for multi-criteria decision making (MCDM) under q-rung orthopair m-polar fuzzy environment are developed. The proposed approaches are suitable to find out an appropriate kind of robotic agri-farming among several kinds of agri-farming. The applications of proposed MCDM approaches are illustrated by respective numerical examples. The comparison analysis of the final ranking and optimal decision in the robotic agri-farming computed by the proposed techniques with some existing MCDM methods is also given to justify the feasibility, superiority and reliability of proposed techniques. Hypothetically, the objective of this artefact may be expanded to establish the algebraic and topological composition like qROmPF-groups, qROmPF-rings, qROmPF-ideals, qROmPF-topology, qROmPF-analysis and qROmPF-graphs. Besides these theoretical aspects, these notions can be extended to solve numerous real world problems and decision making under uncertainty in various fields comprising computational intelligence, cognitive sciences, commerce, business, sociology, econometrics, cleaner production, human resource management, robotics, agri-farming and medical diagnosis. We hope that this article will serve as a foundation stone for the researchers working in these fields.
